# Impact of *ITPA* gene polymorphism for predicting anemia and treatment outcome in HCV infected patients taking Sofosbuvir Ribavirin therapy

**DOI:** 10.1186/s12879-024-09188-1

**Published:** 2024-03-11

**Authors:** Sameen Amjed, Hafiz Ghulam Murtaza Saleem, Sajjad Ullah, Shahzad Latif, Shabana, Junaid Jafar, Ahmad Bilal Waqar

**Affiliations:** 1 University Institute of Medical Laboratory Technology, Faculty of Allied Health Sciences, The University of Lahore, Raiwind Road Campus, Lahore, Pakistan; 2 Medical Laboratory Technology Department, Faculty of Rehabilitation and Allied Health Sciences, RIPHAH International University, Gulberg Campus, Lahore, Pakistan; 3 Gastroenterology Department, Akthar Saeed Medical and Dental College, Lahore, Pakistan; 4 Department of Microbiology and Molecular Genetics, University of the Punjab, Lahore, Pakistan; 5 Specilized Health Care and Medical Education Department, Lahore, Punjab Pakistan

**Keywords:** ITPA, HCV, Ribavirin, Polymorphism, Anemia

## Abstract

**Background:**

Globally, 80 million people are suffering from chronic Hepatitis C virus (HCV) infection. Sofosbuvir ribavirin-based anti-HCV therapy is associated with anemia and other adverse effects. Polymorphisms of Inosine triphosphatase (*ITPA*) gene may cause functional impairment in the Inosine triphosphate pyrophosphatase enzyme, resulting in enhanced sustained viral response (SVR) and protection from ribavirin-associated anemia in patients on therapy. The study objective was to investigate the effect of Inosine triphosphatase gene polymorphism on SVR achievement, hemoglobin decline and ribavirin dose reduction in patients on therapy.

**Methods:**

This prospective cohort study was of 170 hepatitis C infected patients received 6-month sofosbuvir ribavirin therapy. Patient viral load, reduction in ribavirin amount, liver function test, and complete blood count were noted monthly. Inosine triphosphatase variants rs1127354 and rs7270101 were assessed through the restriction fragment length polymorphism and confirmed using Sanger sequencing. The impact of polymorphism on cumulative reduction of ribavirin, and anti-HCV therapy outcome were studied.

**Results:**

A total of 74.3% of patients had *ITPA* rs1127354 CC genotype, 25.7% were CA and AA 0%. The frequency of *ITPA* genotype rs7270101-AA was 95%, AC 5%, and CC was 0%. *ITPA* rs1127354-CA had a notably positive impact on SVR achievement with a zero-relapse rate. *ITPA* rs1127354-CA genotype was significantly (*P* ˂0.05) protective against ≥ 2 g/dl Hb reduction from baseline to 1st, 2nd and 6th months of therapy. During treatment, Hb reduction ≥ 10 g/dl was frequently observed in rs1127354-CC genotype and rs7270101-AA genotype patients. Ribavirin dose reduction was significantly (*P* ˂0.05) high in rs1127354-CC genotype as compared to genotype CA whereas no significant difference was observed in ribavirin dose reduction in rs7270101 AA and non-AA genotype. Patient baseline characteristics such as age, body mass index, rs1127354-CC genotype, and baseline Hb were significantly associated with significant Hb reduction.

**Conclusion:**

Pretreatment evaluation of *ITPA* polymorphism can be a diagnostic tool to find out patients at risk of anemia and improve treatment adherence. ITPA genotype rs1127354-CA contributes to improved compliance with ribavirin dose and protects against hemoglobin decline in HCV patients while taking ribavirin-based therapy. However, *ITPA* rs1127354, rs7270101 polymorphism have no significant impact on SVR achievement.

## Background

HCV is an important cause of liver-related morbidities and patient death. Pakistan ranks 2nd among countries with approximately 11 million HCV reported cases with day-by-day increasing burden. Around 6% of the Pakistani population is HCV-infected. HCV genotype-3 is the most prevalent in Pakistan followed by HCV genotypes 1 and 2 [1, 2]. In the past, primarily interferon ribavirin combination antiviral regimens were effectively in use against HCV in Pakistan. They were reported as having good therapy outcomes but a great number of HCV-infected patients do not achieve sustained viral response (SVR) and also experience severe adverse effects [3]. Particularly ribavirin is reported to cause hemolytic anemia. Ribavirin-induced hemolytic anemia is the cause of ribavirin dose reduction and therapy discontinuation in 10–20% of patients which is also reported to impact antiviral therapy outcomes. The continuous advancement in the discovery of antiviral agents targeting various stages of HCV life cycle is leading towards effective interferon free direct acting antiviral (DAA) therapy [4–6]. The arrival of DAA along with ribavirin against HCV infection resulted over 90–95% SVR rates along with lesser side effects [4, 7].

Treatment of chronic HCV infection has also shown significant challenges including intolerance to standard therapies and low viral clearance. Identifying the factors that can contribute to adverse therapy outcomes and finding those individuals who are likely to get the benefit of treatment have been under study for a long time [8]. Recently, Nonstructural protein 5B (NS5B) inhibitor Sofosbuvir has been permitted at a discounted price for Pakistan; it is currently in effective use and exhibited good SVR rates. Sofosbuvir combination with ribavirin is used solo or combined with interferon for the treatment of different HCV genotypes [9]. Hemolysis is the most significant ribavirin dose-dependent adverse event. Host genetic factors can help in the prediction and modification of therapy, especially concerning the duration and possible benefit of the addition of ribavirin [10]. In HCV patients several *ITPA* gene single nucleotide polymorphisms (SNPs) have been reported to predict anemia while on therapy [11, 12].

The inosine triphosphate pyrophosphatase *(ITPase*) enzyme is encoded by *ITPA* gene, this *ITPase* enzyme is involved in purine metabolism. This *ITPase* enzyme converts inosine triphosphate (ITP) to inosine monophosphate (IMP) and other potential substrates including xanthosine triphosphate (XTP) and deoxyinosine triphosphate (dITP). The *ITPase* is crucial to avoid intracellular buildup of rogue nucleotides ITP, dITP, and XTP which otherwise might be misleadingly incorporated into DNA and RNA producing genetic instability [13]. *ITPase* enzyme hydrolyzes ITP to IDP and IMP therefore, low levels of *ITPase* would lead to a relative abundance of ITP. Inside RBCs, ribavirin causes decreased Na/K transmembrane pump activity, membrane instability, and hemolysis by lowering ATP levels. The increased *ITPA* amount would help in restoring ATP levels as a substitute in place of ATP, in case of levels depleted by ribavirin, and protect from ribavirin-induced hemolytic anemia. The clinical utility of assessing *ITPA* genes includes identifying those individuals who are at the greatest risk of developing anemia, will help the patients to receive planned ribavirin dose with minimum side effects and identifying those who might tolerate a higher dose of ribavirin during treatment [8, 13–15].

Studies on individuals of European, African, and Japanese population has shown an association between *ITPA* variants with ribavirin-induced anemia [14]. Rembeck in 2015 reported significant associations between the likelihood of achieving HCV clearance and reduced *ITPase* enzyme [10]. In an Italian study, HCV-infected patients with reduced *ITPase* activity presented better SVR rates when treated with pegylated interferon and ribavirin. This association was also reported in HCV genotype-1 infected Japanese patients [16]. Study from Japan found that *ITPA* rs1127354 polymorphism was associated with ribavirin-related Hb decline, but no significant impact of *ITPA* polymorphism was found on SVR achievement rate [17]. A genome-wide association study also exhibited a strong association between *ITPA* polymorphisms and ribavirin-induced hemolytic anemia [11]. Two ITPA SNPs rs1127354 and rs7270101 reported a protective role against anemia due to ribavirin in HCV patients on therapy but were not associated with antiviral therapy outcomes [18–20]. Ribavirin is used as a supportive anti-HCV drug to reduce treatment duration, increase treatment outcome although produce adverse effects mainly ribavirin associated anemia. Determination of *ITPA* gene variants is a good predictive strategy of ribavirin-induced anemia, thus genotyping before starting antiviral treatment can work as a biomarker and play a prognostic role in clinical practice for pretreatment screening, therapy individualization, improving treatment outcome and reduction of ribavirin-associated Hb decline [15].

This study is intended to find out the impact of *ITPA* genetic polymorphisms on treatment outcomes in HCV-infected patients as a prognostic factor in improved management of patients. Pretreatment determination of *ITPA* polymorphism will help in early identification of anemia predisposition and its associated complications, which may have various implications for HCV affected individuals. The literature lacks studies of Pakistani population exploring the effect of *ITPA* polymorphism as a predicting factor for anemia development in HCV patients taking sofosbuvir ribavirin combination therapy. *ITPA* gene testing may work as a pharmacogenetic diagnostic tool to predict drug-induced adverse events, particularly for high-risk patients.

## Methods

### Study design and patient selection

This prospective cohort study was conducted in 2020 at the regional hepatitis center in Lahore after taking ethical approval reference number (UOLFAHS/565). A total of 170 HCV patients were assessed after taking ethical approval and consent was taken to participate. Patients with an age range of 18–60 years infected with HCV genotype 3a, of both genders, and who were new to treatment (no history of previous treatment for HCV) were included. The patients below 18 years of age, pregnant females, patients with a previous history of HCV treatment, patients found with co-infection of hepatitis B virus, Human immune deficiency virus, patients with liver cirrhosis, leukemia, alcoholics, and other comorbidities were excluded from this study. The patients were enrolled to be treated with sofosbuvir nucleoside analog inhibitor of HCV NS5B polymerase 400 mg once daily and weight-based ribavirin (for ˂75Kg weight 100 mg/day and > 75Kg body weight 1200 mg/day) for 6-months. Written informed consent to participate was taken. After obtaining approval from the ethical committee the HCV patients receiving DAA therapy were invited to participate and demographic data was also recorded.

### Efficacy assessments

At baseline (before receiving DAA therapy), liver ultrasound was performed. Patients were screened by enzyme-linked immunosorbent assay (ELISA) for hepatitis B virus, Human immune deficiency virus, and hepatitis C virus. Tests including Hepatitis C viral load (Real-time PCR), liver function tests, and complete blood count (CBC), were performed monthly (1–6 months).

### Study endpoints

For viral end points patients with an undetectable virus at the end of treatment were described to have achieved a CVR complete viral response. Relapse was defined as patients after achieving CVR successfully presented detectable HCV RNA during after-treatment follow-up. Only patients with the undetectable viral RNA at CVR and at follow-up 24 weeks after completion of treatment were considered to have achieved SVR (Sustained viral response). The rate of SVR achievement was recorded as Per protocol analysis (PPA) means those patients were included in the study who adhered to anti-viral therapy till the finishing point and their post-treatment SVR status was assessed [21]. A decline in hemoglobin levels in HCV patients after using DAA (sofosbuvir ribavirin) treatment was also noted [5].

### Determination of *ITPA* polymorphism

EDTA anticoagulated blood was collected aseptically and was used for DNA extraction following the technique described previously [22]. *ITPA* gene amplification was performed with conventional PCR using (Thermo Scientific by Thermo Fisher) PCR master with 5–10 ng/µl DNA. Forward primer F-5′‐AGATGGGCAGCAGAGTTATCG‐3′ and the reverse primer was R‐5′‐AGACAGAGAAATCCAACCATCTTTTAAGAA 3′ [21]. The thermal cycle conditions were as described in our previous study [23].

Following PCR, the *ITPA* genotypes were initially analyzed using RFLP (Restriction fragment length polymorphism). For RFLP, PCR products 213 bp were treated overnight with restriction enzymes Xcel and Mboll to investigate rs1127354 and rs7270101 variants polymorphisms respectively [21]. The *ITPA* genotype results were confirmed through Sanger sequencing and results were analyzed by Chromas version 2.5.0.

### Statistical analysis

Based on the patient’s *ITPA* genotype a comparative analysis of data was performed. Shapiro-Wilk test was used to test the normality of data. Quantitative variables were described as mean ± SD and qualitative variables were reported as frequencies and percentages. Categorical and continuous variables were compared by the Chi-square test (X2) and Mann-Whitney U test respectively. Kaplan-Meier analysis and the log-rank test were used to estimate and compare ribavirin dose reductions between the *ITPA* genotypes. To identify variables associated with Hb reduction ≤ 2 g/dl during treatment Binary logistic regression analysis was used. All tests were two-tailed and performed using the SPSS software version 25.0 and *P* ≤0.05 was considered statistically significant.

## Results

**Fig. 1:**
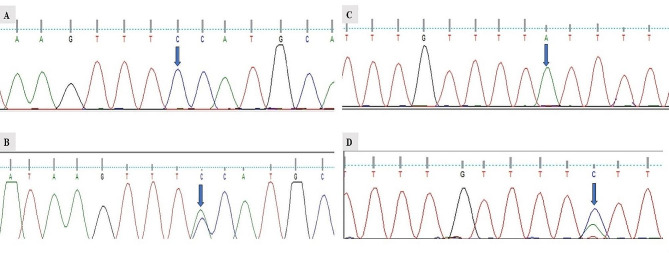
Sequencing chromatogram of *ITPA* polymorphism rs1127354 and rs7170101. (A) Chromatogram of rs1127354-CC genotype. (B) Chromatogram of rs1127354-CA genotype (Missense mutation). Arrow indicates the point where the mutation occurred. (C): Chromatogram of rs7270101-AA genotype. (D) Chromatogram of rs7070101-AC genotype (Splice variant). Arrow indicates the point where the mutation occurred, A, T, G, and C present nucleotide positions as adenine, guanine, cytosine, and thiamine

One fifty out of a total of 170 patients completed 6-month antiviral therapy and were effectively tracked till CVR. Out of which 140 HCV patients remain successfully associated with the study for a further 6 months till SVR. In the case of *ITPA* rs1127354 polymorphism homozygous (CC) and heterozygous (CA) allele fragments of 213 bp and 213, 135,78 bp were observed. While in case of *ITPA* polymorphism rs7170101(AA) allele, and (AC) allele fragments of 213 bp and 213, 173, 40 bp were observed respectively. No homozygous recessive alleles (rs1127354-AA and rs7170101-CC) were found in our study samples. Both *ITPA* variants rs1127354 and rs7270101 data obeyed the Hardy-Weinberg equilibrium. RFLP results were further confirmed using Sanger sequencing. The sequencing chromatogram of *ITPA* polymorphism is presented in Fig. 1.

### *ITPA* SNPs and treatment outcome with Sofosbuvir Ribavirin therapy

**Fig. 2:**
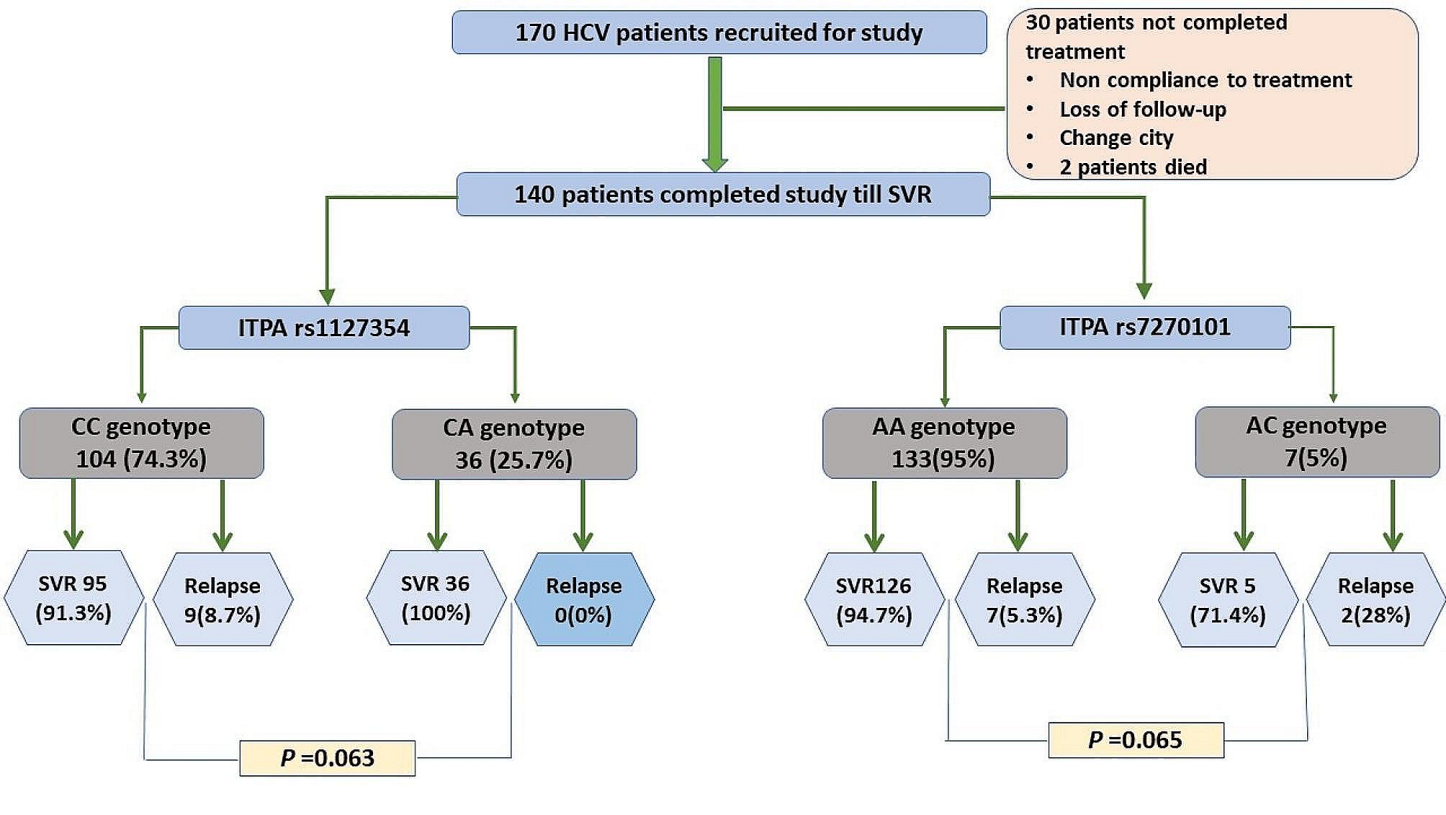
*ITPA* rs1127354, rs7270101 genotype wise presentation of SVR achievers and relapsed HCV patients receiving Sofosbuvir ribavirin. Sustained Viral response (SVR)

Overall, CVR was achieved by 99.3% of HCV patients. Out of 140 patients, 93.6% of patients achieved SVR. An important finding was that 100% of individuals having *ITPA* rs1127354-CA genotype achieve SVR. Anti-HCV treatment outcome on the basis of the patient’s *ITPA* SNP rs1127354 and rs7270101 genotypes is presented in Fig. 2.

The CVR achievement rate was similar between both *ITPA* genotypes rs1127354 and rs7270101. No significant difference was observed between the baseline features of both group patients presented in Table 1. Table 2 presents the demographic profile of HCV patients taking sofosbuvir ribavirin therapy and whether they achieve SVR or relapsed.

**Table 1:** Demographic profile of HCV patients (*ITPA* rs1127354-CC and Non-CC genotype)

Variable	CC genotype Mean ± SD	Non-CC genotype Mean ± SD	P-value
Age	41.3 ± 10.2 (21–60)	42.5 ± 12.2(21–59)	0.415
Gender Male/Female	58(52.3%)/53(74.7%)	24(61.5%)/15(38.5%)	0.280
Body mass index	26.5 ± 4.8 (13.2–37.1)	25.3 ± 3.7(20-34.6)	0.146
Hemoglobin g/dl	13.2 ± 1.1(11-16.1)	13.2 ± 1.3 (11.8–16.1)	0.67
HCV RNA by PCR (IU/ml)	2.8 × 10^6^ ± 5.8 × 10^6^ (7525-32367153)	2.6 × 10^6^ ± 3.9 × 10^6^ (6601-13032163)	0.839
Red blood cell count (10^12^ /L)	4.3 ± 0.3 (3.6–5.3)	4.3 ± 0.4 (3.9–43)	0.89
Hematocrit l/l	39.3 ± 3.4 (34–48)	38.7 ± 3.7(35–49)	0.115
Total leukocyte counts (IU/L)	7.3 ± 2.2 (3.5–15.5)	7.7 ± 2.3 (3.9–13.1)	0.323
Platelets 10^9^ cells/L	224 ± 60 (100–400)	204 ± 66 (100–401)	0.43
Serum Urea mg/dl	28.5 ± 4.4 (19–44)	28 ± 4.7 (21–39)	0.840
Serum creatinine mg/dl	0.7 ± 0.1 (0.5–1.4)	0.7 ± 0.1 (0.5-1.0)	0.890
Total Bilirubin (mg/dl)	0.6 ± 0.2 (0.4–1.5)	0.7 ± 0.4 (0.4–1.9)	0.958
ALT (IU/L)	68 ± 42 (19–225)	72 ± 37 (24–177)	0.342
AST (IU/L)	69 ± 48.1 (22–261)	73 ± 45 (29–201)	0.395

**Abbreviations**: Aspartate amino transferase (AST), Alanine aminotransferase (ALT), Polymerase chain reaction (PCR), Standard deviation (SD)

**Table 2:** Demographic characteristics of patients who achieve SVR and patients who relapsed

Variable		Sustained viral response (SVR) achieved	Relapsed	P-value
	Age	40.9 ± 10.9	46.6 ± 12.3	0.462
Gender	Male	71 (54.2%)	5 (55.6%)	0.937
	Female	60 (45.8%)	4 (44.4%)	
Genotype rs1127354	CC	95 (72.5%)	9 (100%)	0.06
	CA	36 (27.5%)	0%	
Genotype rs7270101	AA	126 (96.2%)	7 (77.8%)	0.01
	AC	5 (3.8%)	2 (22.2%)	

### *ITPA* genotype and liver ultrasound

A total of 28 (41%) patients had fatty liver whereas normal liver architecture was found in 50% of patients. There was no significant difference between the liver architecture of patients based on patient gender and *ITPA* rs1127354 genotype.

### *ITPA* SNP and reduction in Hb concentration

**Fig. 3:**
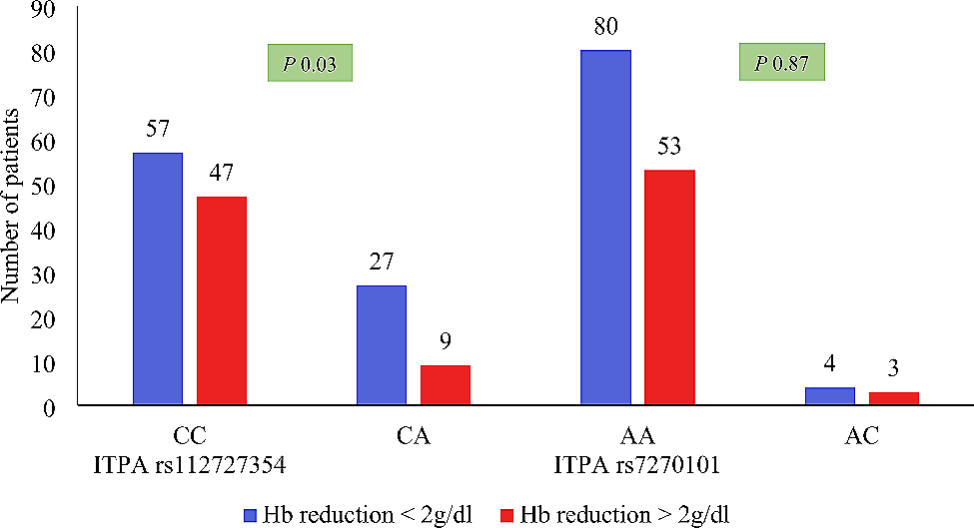
Hb reduction ≥ 2 g/dl from baseline to 1st month according to *ITPA* polymorphism. The height of bars presents the number of HCV infected patients with belong to each *ITPA* genotype experience hemoglobin reduction after weight-based ribavirin (for ˂75Kg weight 100 mg/day and > 75Kg body weight 1200 mg/day) was given

When pretreatment Hb levels were compared to 1 month post treatment Hb levels, 89 (59.3%) HCV-infected patients showed ˃2 g/dl Hb reduction, but only 61 (40.7%) persons showed ˂ 2 g/dl Hb reduction. Significant Hb reduction as compared to baseline Hb was found in *ITPA* rs1127354 CC and CA genotypes but this difference was not significant in *ITPA* rs7270101 genotypes Fig. 3.

**Fig. 4:**
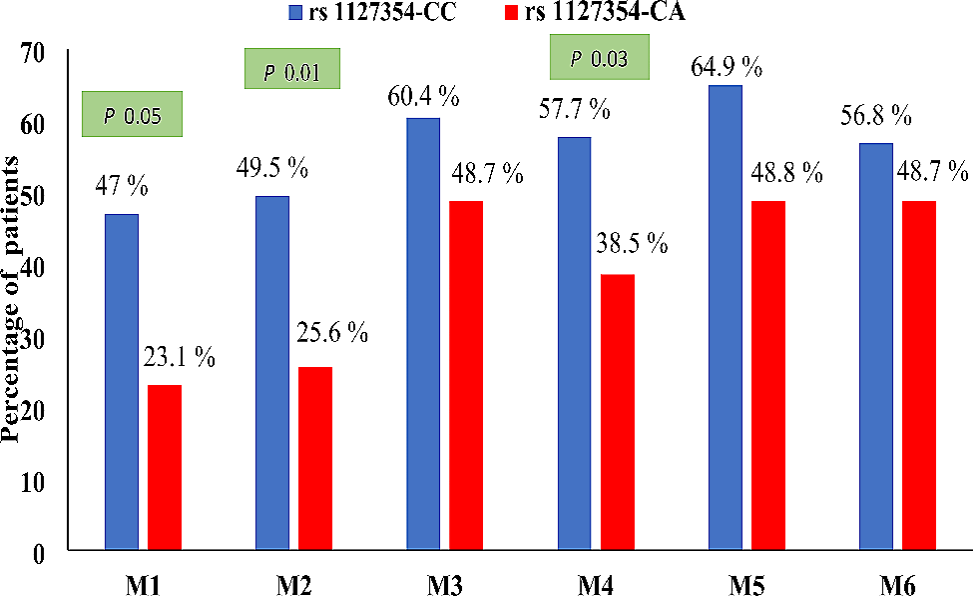
Month wise ≥ 2 g/dl Hb reduction with reference of *ITPA* rs11273547 CC and CA genotype. Months are presented as M1, M2, M3, M4, M5, M6. The bars present percentage of patients with each *ITPA* genotype experience hemoglobin reduction. Significantly high number of patients with ITPA SNP rs1127354-CC experience ≥ 2 g/dl Hb reduction at months M1, M2, and M4 mentioned by *P* value

Patients on DAA therapy were observed monthly for Hb reduction ≥ 2 g/dl. Significantly high (*P* = 0.05, *P* = 0.01, *P* = 0.039) number of *ITPA* SNP rs1127354-CC patients experience ≥ 2 g/dl Hb reduction at months 1,2, and 4 during 6-month sofosbuvir ribavirin therapy with reference to baseline Hb levels as compared to rs1127354-CA (Fig. 4).

Reduction in Hb less than ≤10 g/dl was found in 35 (23.3%) HCV patients during DAA therapy. Out of which 29 (26.1%) belong to the *ITPA* rs1127354-CC group and 6 (15.4%) belong to the CA group. Whereas 22(15.5%) of patients with *ITPA* rs7270101 AA genotype had Hb less than 10 g/dl and only 1 (12.5%) patient with rs7270101 AC had Hb less than 10 g/dl.

### The mean difference in Hb concentration

**Fig. 5:**
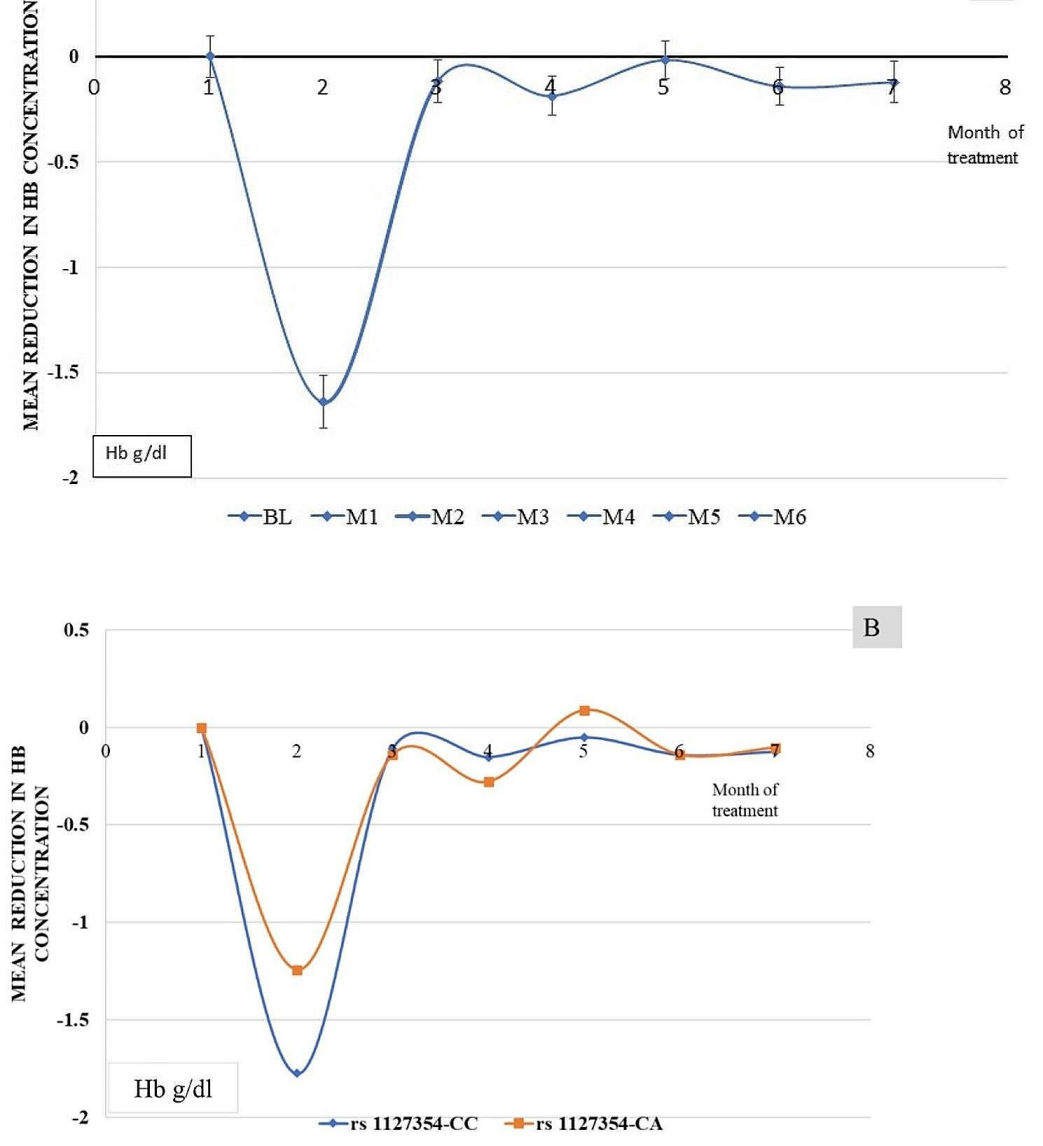
Changes in mean hemoglobin concentration of HCV infected patients during Sofosbuvir and Ribavirin treatment. (A) Changes in mean Hb concentration of overall patients. (B) Changes in mean Hb concentration with reference of ITPA rs1127354 CC and CA. Base line (BL), Month of treatment (M1, M2, M3, M4, M5, M6)

The mean Hb concentration of HCV patients at baseline was 13.2 ± 1.2. Month-wise difference of means of Hb concentration as compared to baseline Hb was calculated. A significant reduction in Hb concentration in the 1st month of antiviral therapy was observed with reference to baseline Hb concentration Fig. 5A. The mean difference of Hb concentration when plotted on the basis of *ITPA* SNP, reduction in Hb concentration was observed from baseline was high in the *ITPA* rs117354-CC genotype as compared to CA Fig. 5B.

### Ribavirin dose reduction and *ITPA*-SNPs

**Fig. 6:**
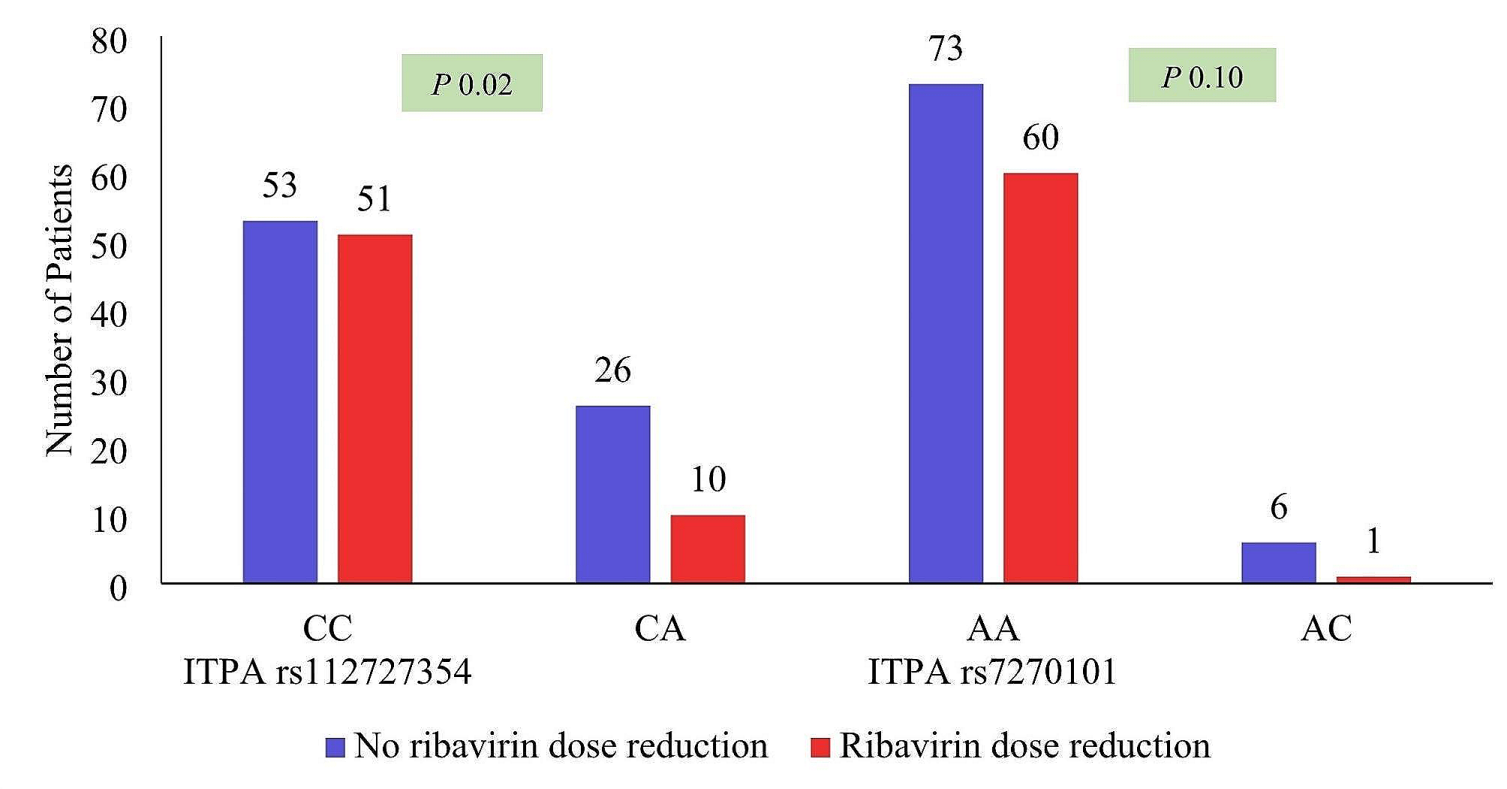
Ribavirin dose reduction relative to *ITPA* polymorphism in HCV patients taking Sofosbuvir ribavirin therapy. The bars present number of patients experience ribavirin dose reduction by *ITPA* polymorphism rs1127354 CC and CA genotype and *ITPA* rs7270101 AA and Ac genotype

Ribavirin doses were reduced in response to Hb decline, ribavirin amounts were reduced in total to 64 (42.7%) doses relative to the planned ribavirin dose as necessary according to treatment guidelines. Ribavirin dose reduction was frequently higher in *ITPA* rs1127354-CC individuals. However no significant difference was observed in ribavirin dose reduction in *ITPA* rs7270101 AA and Non-AA genotype Fig. 6.

**Fig. 7:**
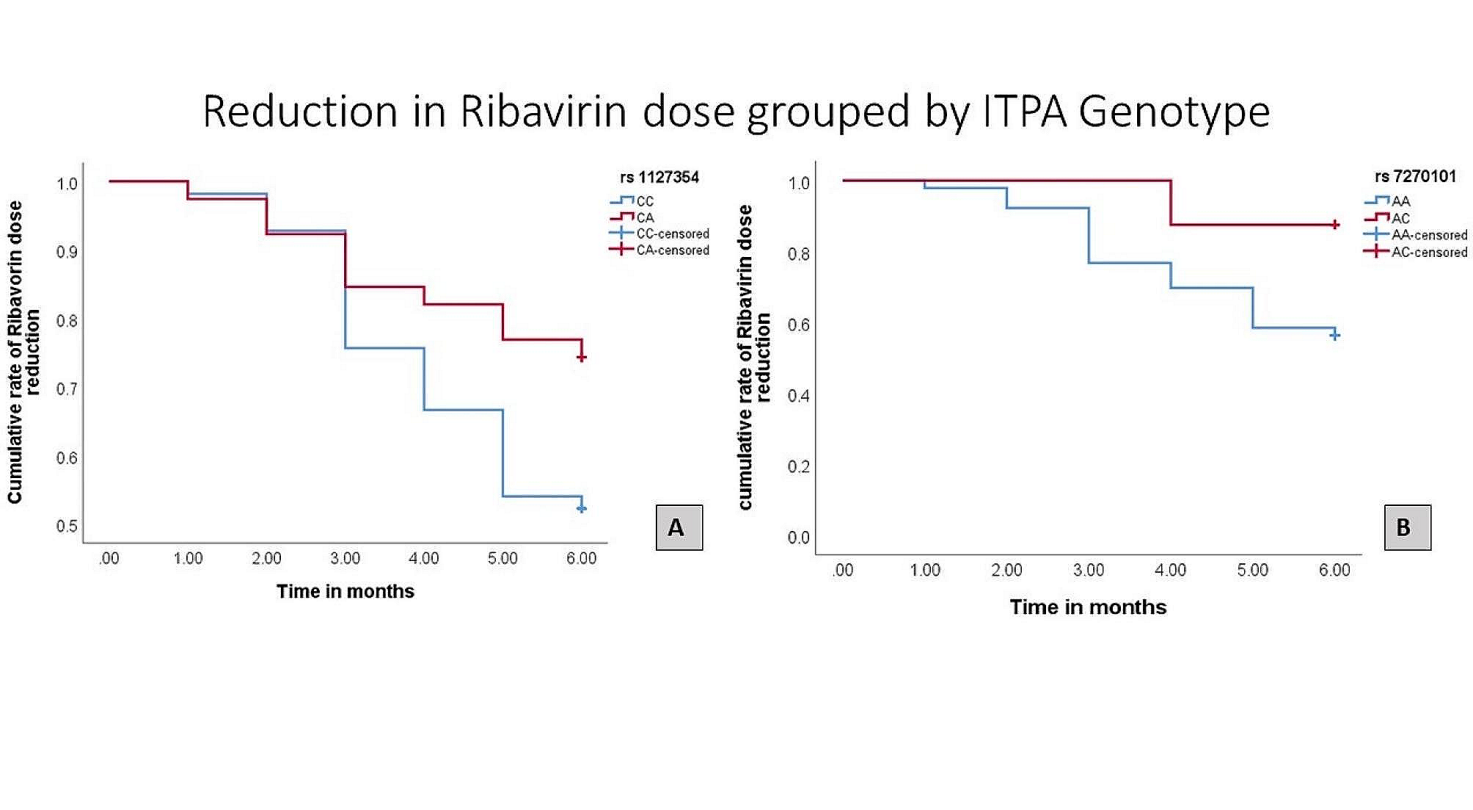
Ribavirin dose reduction on basis of *ITPA* genotype (Kaplan-mere curve). (A) Ribavirin reduction in rs1127354-CC & CA genotype. (B) Ribavirin dose reduction in *ITPA* rs7270101 AA and AC genotype

Survival analysis of data presented that there was a significant difference *P* ≤0.05 between month-wise ribavirin dose reduction in *ITPA* rs1127354-CA and CC genotype. Contrary to this no significant difference was observed in month-wise ribavirin dose reduction *ITPA* rs7270101 genotype AA and AC Fig. 7.

### Prognostic factors linked with ≥ 2 g/dl Hb reduction with respect to baseline Hb value

To determine prognostic factors linked with ≥ 2 g/dl Hb reduction with respect to baseline Hb. Binomial logistic regression analysis was used. The (Nagelkerke R^2^) was 31.9%. It was observed that HCV patient age, body mass index, baseline Hb, and patients’ *ITPA* genotype rs1127354-CC were significantly (*P-value* < 0.05) associated with a reduction of ≥ 2 g/dl Hb level as compared to baseline Hb value (Table 3).

**Table 3:** Pretreatment characteristics of HCV patients associated with Hb reduction ≥ 2 g/dl compared to pretreatment Hb value

Pretreatment characteristics of HCV patients	Odd ratio (95% CI)	P-value
Age of patient	1.07 (1.03–1.11)	0.00
Patient gender	0.59 (0.21–1.68)	0.33
*ITPA* rs1127354	4.86 (1.79–13.2)	0.00
*ITPA* rs7270101	0.63 (0.11–3.44)	0.59
Body mass index	0.89 (0.81–0.97)	0.01
Viral load	1.00 (1.00–1.00)	0.34
White blood cell count	1.05 (0.87–1.26)	0.60
Red blood cell count (10^12^ /L)	0.00 (0.00- 45.6)	0.14
Hemoglobin	71.9 (0.49–10,558)	0.04
Platelet	0.99 (0.98–1.0)	0.15
Aspartate Aminotransferase	1.013 (0.99–1.03)	0.16
Alanine aminotransferase	0.98 (0.96–1.00)	0.18
Bilirubin (Total)	0.24 (0.04–1.25)	0.09
Serum creatinine	0.43 (0.01–9.65)	0.59
Serum urea	0.92 (0.83–1.02)	0.11

### Reduction in Hb concentration due to Ribavirin-based therapy and impact on SVR achievement

Out of all 9 patients who not achieve SVR analysis of data presented that most of the patients (6 out of 9) had Hb reduction > 2 g/dl Hb when 1 month post treatment Hb levels were compared to pretreatment Hb levels but the difference was not significant (*P* = 0.09). Moreover, no significant difference was observed *P* = 0.53 between the incidence of anemia throughout the treatment in patients who achieve SVR and those who relapsed.

### Baseline prognostic factors of HCV patient linked with SVR achievement

To determine the baseline prognostic factors linked with SVR achievement (antiviral response) binomial logistic regression analysis was used. The result of analysis presented (Nagelkerke R^2^) was 28.7% but none of the baseline variable was found significantly associated with SVR achievement or relapse in our under-study population (Table 4).

**Table 4:** Pretreatment characteristics of HCV patients associated with SVR achievement

Pretreatment characteristics of HCV patients	Odd ratio (95% CI)	P-value
Age of patient	1.07 (0.98–1.16)	0.10
Patient gender	2.07 (0.24–17.5)	0.50
*ITPA* rs1127354	0.001 (0.00–0.00)	0.99
*ITPA* rs7270101	26.5 (1.8–389)	0.09
Body mass index	1.10 (0.91–1.33)	0.31
Viral load	1.00 (1.00–1.00)	0.92
White blood cell count	0.93 (0.66–1.32)	0.70
Hemoglobin	0.76 (0.30–1.9)	0.57
Platelet	0.99 (0.98–1.01)	0.61
Aspartate Aminotransferase	0.99 (0.96–1.02)	0.87
Alanine aminotransferase	1.01 (0.98–1.04)	0.45
Bilirubin (Total)	0.28 (0.010–8.30)	0.46

## Discussion

The recent development of DAAs for the treatment of HCV patients helped to achieve high SVR rates and improved patient safety. DAA in combination with ribavirin is reported to cause anemia which could restrict therapy. Several reasons, including patient *ITPA* genotype, have been reported to be significantly associated with ribavirin-induced anemia during treatment [24]. Literature observed with studies lacks in exploring the effects of DAA with ribavirin on Hb reduction levels, concerning *ITPA* polymorphisms from the Pakistani population. The current study found that 74.3% of HCV patients were having *ITPA* rs1127354 CC genotype and the frequency of *ITPA* heterozygous allele rs1127354-CA was 25%, while 95% of patients had rs7270101-AA genotype frequency and rs7270101 genotype-AC was only 5%. The findings of the present study are similar to the study from Japan reported frequency of rs1127354 CC genotype was 75% and 25% had a non-CC % [25]. An Italian study stated that *ITPA* genotypes at rs1127354 were 90% CC, 8% CA, and 1.2% AA. Whereas *ITPA* rs7270101 genotypes were 78% AA, 19.3% AC, and 1.6% CC [26]. Kim et al. conducted a study in Korea and found that 81% of patients had genotype rs1127354-CC and 18% were non-CC [27]. The study from Peshawar Pakistan reported that *ITPA* rs1127354 CC genotype was 60% and 33% were genotype CA, and only 2.6% of HCV patients had AA genotype. While all patients presented *ITPA* rs7270101-AA genotype [28]. Contrary to our study from Iran found the genotypic frequency of *ITPA* rs1127354 was 79% CC, 20% CA, and 1% AA and *ITPA* rs7270101 was 80% AA, 19% AC and 1% CC [21].

Japanese studies reported a correlation of ribavirin associated anemia incidence and *ITPA* SNP rs1127354 polymorphism and ribavirin dose reduction but was not linked to antiviral therapy outcome and no polymorphism of gene at *ITPA* SNP rs7270101was observed. Pretreatment characteristics including patient age, baseline hemoglobin, and *ITPA* rs1127354-CC genotype were independently associated with the development of anemia [5, 12]. In HCV patients a real-world study on Caucasian patients showed that *ITPase* deficiency was associated with protection from anemia in HCV patients having pegylated interferon and ribavirin treatment. *ITPase* deficiency would help in ribavirin dose reduction to a lesser extent and also support to use of less supportive agents such as blood transfusions and erythropoietin against anemia as treatment efficacy is reported to be hampered by dose reductions [14]. In the present study *ITPA*, heterozygous allele rs1127354-CA has been found to protect significantly against Hb reduction. In HCV patients the reduction of hemoglobin ≥ 10 g/dl was frequently observed in *ITPA* wild (rs1127354-CC) genotypes. Hb reduction ≥ 2 g/dl with respect to pretherapy Hb level was significantly associated with variables like age of the patient, BMI, having rs1127354-CC, and baseline Hb level. Overall good 93% rates of SVR with sofosbuvir ribavirin combination therapy were observed, and notably, no patient with *ITPA* genotype rs1127354-CA presented as relapsed after achieving SVR. However, there was no significant impact of the *ITPA* genotype of the patient on SVR achievement. Similar findings of a study from Islamabad (Pakistan) SVR rate was 96% [29], and a study from Peshawar (Pakistan) reported age was a key factor in achieving SVR in HCV subjects [30]. Thompson et al. described that *ITPA* rs7270101 & rs1127354 variants were linked with anemia due to ribavirin. Minor alleles of both polymorphisms rs1127354 & rs7270101 were protective against anemia but presented any positive impact on SVR rate improvement [26]. Kim et al. reported from Korea that *ITPA* non-CC genotypes at rs1127354 are associated with protection against anemia due to ribavirin during ribavirin & interferon treatment in HCV-infected individuals. However, no association was found with SVR rate improvement [27]. Urabe et al. reported that *ITPA* CC genotype HCV-infected patients frequently experienced anemia as compared to *ITPA* CA/AA genotype individuals [31].

In a study from Egypt conducted on chronic HCV receiving pegylated-interferon & ribavirin treatment and *ITPA* rs1127354-CC genotype was linked with hemoglobin reduction at 1st month of therapy. Other factors such as female gender and lower baseline Hb were associated with anemia at week 4 of treatment. *ITPA* genotype-CC patients experienced hemoglobin reduction in response to which ribavirin dose was reduced more frequently. There was no impact of *ITPA* polymorphism on SVR achievement [32]. A study conducted in Peshawar Pakistan also reported that *ITPA* rs1127354 minor allele-A is protective against ribavirin-induced anemia during interferon ribavirin anti-HCV treatment [28]. Another study from Karachi Pakistan reported around 16.0% of on-therapy patients had a ribavirin dose reduction due to adverse effects of anemia. A total of 59.4% developed anemia, and 23.6% had severe anemia. The severity of anemia was related to the patient’s age and baseline hemoglobin levels [33].

In the current study ribavirin dose reduction in response to adverse effects was more prominent in HCV-infected patients with *ITPA* rs1127354-CC genotype but not in CA genotype whereas this difference was not observed in *ITPA* SNP rs7270101 because only 5% of patients were showing polymorphism in the study population. Likewise, Nemr et al. reported that *ITPA* rs1127354 polymorphism was associated with ribavirin-based Hb reduction in Egyptian patients receiving anti-HCV treatment. Hemoglobin decline and ribavirin dose reduction were higher among *ITPA* rs1127354 CC individuals [34]. Azakami et al. studied the effect of *ITPA* variants on therapy response. A rapid reduction in Hb levels was observed in rs1127354-CC genotyped individuals and comparatively slow Hb reduction was observed in non-CC patients. Cumulative ribavirin dose reduction was also higher in rs1127354-CC than non-CC individuals but no influence of *ITPA* variants was observed on treatment outcome [35]. On another hand, Hwang et al. reported that *ITPA* rs7270101 and rs1127354 are protective against ribavirin-associated anemia in HCV patients on therapy but have no influence on therapy outcome. In the Asian population, *ITPA* rs7270101 is excluded because no such variants have been reported in the international HapMap project database [36].

### Conflict

ing this, a conclusion statement of meta-analysis of 2020 found patients with *ITPA* rs1127354-CC polymorphism are more likely to be involved in the development of hemolytic anemia and decreased rate of SVR. Literature learned from a few studies discussed the effect of rs7170101 polymorphism. They also found statistical evidence of an association between ribavirin dose reduction and *ITPA* polymorphism [4]. A real-life study was conducted on HCV genotype 2 patients, and it was observed that regardless of previous antiviral treatment history sofosbuvir-based treatment schedules were associated with good tolerability and SVR achievement rates, without a significant impact, on treatment ribavirin dose reductions [37]. The results of present study determine that ribavirin based DAA therapy has good SVR achievement rate in our population and pretreatment evaluation of *ITPA* polymorphism can help to reduce the risk of developing anemia due to ribavirin. The study’s drawback was that more attention may have been paid to additional variables or confounders that might have an impact on the findings. Nevertheless, the number of patients who completed the study was modest that may affect the precision of our estimates and the ability to generalize our findings to the broader population, but then again, the insights gained from present study provide valuable information on the risk factors associated with anemia in the context of HCV infection in the Pakistani population. These findings can serve as a foundation for larger-scale studies in our population in the future.

## Conclusion

The current study concludes that sofosbuvir ribavirin therapy provides good SVR rates achievement in HCV but is associated with the risk of certain adverse effects, notably ribavirin-associated anemia. We found patients with *ITPA* genotype rs1127354-CC are significantly at higher risk of developing ribavirin-associated anemia as compared to patients with a non-CC genotype that leads to less adherence to ribavirin dose but no statistically significant impact of *ITPA* genotype has been found on SVR achievement. These findings highlight the importance of pretreatment genotype evaluation of *ITPA* which can be used as a pharmacogenetic diagnostic tool in clinical practice to find out risky patients and individuals who can benefit from ribavirin. It will also help in individualization and management of ribavirin dose to get maximum benefit. The study limitation was that a very small number of rs7270101 heterozygous allele patients were found which makes it difficult to monitor its effect on Hb reduction and treatment outcome. We acknowledge that larger studies to further explore the impact of ITPA polymorphism on ribavirin based anti-HCV therapy tolerability and treatment outcome are warranted to confirm and extend our observations.

## Data Availability

All data is available with the corresponding author and can be accessed on request. The datasets generated and/or analyzed during the current study are available on the Figshare with citation Amjed, Sameen (2023). ITPA SNP data.xlsx. figshare. Dataset. https://doi.org/10.6084/m9.figshare.24847809.v3.

## Abbreviations

ITPA: Inosine triphosphatase
HCV: Hepatitis C virus
CVR: complete viral response
SVR: sustained viral response
DAA: Direct acting antiviral
SNP: Single nucleotide polymorphisms
IMP: Inosine monophosphate
XTP: Xanthosine triphosphate
dITP: Deoxyinosine triphosphate
CBC: Complete blood count
RFLP: Restriction fragment length polymorphism
